# The COVID-19 Pandemic and Smoking Cessation—A Real-Time Data Analysis from the Polish National Quitline

**DOI:** 10.3390/ijerph19042016

**Published:** 2022-02-11

**Authors:** Paweł Koczkodaj, Magdalena Cedzyńska, Irena Przepiórka, Krzysztof Przewoźniak, Elwira Gliwska, Agata Ciuba, Joanna Didkowska, Marta Mańczuk

**Affiliations:** 1 Cancer Epidemiology and Primary Prevention Department, Maria Sklodowska-Curie National Research Institute of Oncology, 02-781 Warsaw, Poland; magdalena.cedzynska@pib-nio.pl (M.C.); irena.przepiorka@pib-nio.pl (I.P.); krzysztof.przewozniak@pib-nio.pl (K.P.); elwira.gliwska@pib-nio.pl (E.G.); agata.ciuba@pib-nio.pl (A.C.); joanna.didkowska@pib-nio.pl (J.D.); marta.manczuk@pib-nio.pl (M.M.); 2 Department of Food Market and Consumer Research, Institute of Human Nutrition Sciences, Warsaw University of Life Sciences-SGGW, 02-776 Warsaw, Poland; 3 Department of Social Medicine and Public Health, Doctoral School, Medical University of Warsaw, 02-007 Warsaw, Poland

**Keywords:** tobacco, smoking, smoking cessation, COVID-19, pandemic, Poland

## Abstract

Since the outbreak of the COVID-19 pandemic, tobacco research has delivered new evidence on the harmfulness of smoking in the context of SARS-CoV-2 infection and the course of the COVID-19 disease. More and more research proves that smoking is an important risk factor contributing to increased risk of mortality among COVID-19 patients. The aim of this study was to assess whether and how the COVID-19 pandemic impacted decisions about quitting smoking. A total of 4072 records of anonymized data were obtained from the Polish National Quitline. Between 15 April 2020 and 31 May 2021, the callers were asked about the COVID-19 pandemic and its influence on their decisions on smoking continuation or cessation. Our results indicate that smokers are very receptive to communication concerning COVID-19 and smoking risk. This phenomenon can possibly be connected to the immediate potential health consequences of smoking and COVID-19 virus infection. Results may indicate that putting emphasis on arguments combined with short-term health consequences of smoking may result in better outcomes in smoking cessation. There is a need for further and constant education on tobacco-related health harm. Our results showed that an irregular and mass communication on health consequences may result in high effectiveness in smoking cessation.

## 1. Introduction

Smoking remains one of the biggest public health challenges worldwide. According to World Health Organization (WHO) data, about 8 million people die annually because of tobacco-related diseases. Moreover, about 1.2 million deaths are related solely with passive smoking [1]. The annual economic cost of consuming tobacco products worldwide is estimated at around 2 billion USD (estimates based on 2016 data). Most of these costs are related to loss of productivity due to illness or death, as well as treatment of tobacco-related diseases. In Poland, the tobacco-related death rate for men is one of the highest in the world (Poland is in the group of countries where 25% or more of male deaths are related to tobacco use) [2]. Additionally, Poland has one of the highest values of the tobacco-related DALY (Disability Adjusted Life-Years) indicator among European countries. This indicator provides information on the number of years of life lost as a result of bodily injury or premature death—in this case—caused by tobacco use [3].

Cigarette smoking is the most documented cause of disease ever studied [4]. Up to 90% of lung cancer cases among men and 70–80% among women are directly connected with smoking [5]. Lung cancer is just one of the many health consequences of exposure to tobacco smoke. Others include bladder cancer; cancer of the kidneys; liver; pancreas; stomach; colon and rectum; cervix; acute myeloid leukemia; cardiovascular diseases; chronic obstructive pulmonary disease (COPD); strokes; blindness; osteoporosis; decreased fertility; fetal malformations, prematurity, and miscarriages; among many others [6,7]. Among young people, in addition to the risk of addiction itself, nicotine negatively affects the development of the nervous system by disrupting cognitive functions, making it difficult to concentrate and learn [8].

Since the beginning of 2020, when the COVID-19 pandemic outbreak took place, studies have delivered new evidence on the harmfulness of smoking—this time in the context of SARS-CoV-2 infection and the course of the COVID-19 disease. More and more research proves that smoking is an important risk factor contributing to increased risk of mortality among COVID-19 patients [9]. Research has shown that any history of smoking in the individual’s life is combined with a substantially higher vulnerability to severe course of the COVID-19 disease, as well as much worse prognosis in the event of hospital treatment [10]. Moreover, smoking is a risk factor for progression of the COVID-19. Those who have never regularly smoked (or “never-smokers”) demonstrate much lower risk in this context compared to people who have ever smoked cigarettes [11]. Many of the published tobacco studies were highlighted by Polish media, unintentionally sparking a spontaneous, nationwide anti-tobacco campaign. A reflection of this phenomenon was visible in the evolving characteristic of callers as well as in the questions they posed during consultations with specialists from the Polish National Quitline.

Following the observed changes, we decided to study whether the pandemic outbreak could potentially impact smoking prevalence in Poland. The aim of this study was to assess whether and how the COVID-19 impacted decision-making about quitting smoking, taking into account socioeconomic context (e.g., smoking status, sex, and age group).

## 2. Materials and Methods

Anonymized data were collected by the Polish National Quitline (operating at the Maria Sklodowska-Curie National Research Institute of Oncology in Warsaw, Poland, continuously since 1996), which provides help in smoking cessation six days a week (Monday–Saturday). In addition to the standard questions (such as age, gender, place of residence, smoking status, motivation to quit, etc.), those who called between 15 April 2020 and 31 May 2021 were also asked about the COVID-19 pandemic and its influence on their decisions on smoking continuation or cessation. These additional questions were asked regardless of whether the consultations were proactive or reactive. The following questions were included in the additional questionnaire:

1. Has the COVID-19 pandemic impacted your decision on quitting smoking?
2. How has the current pandemic situation impacted your decision on quitting smoking?
3. Do you think that smoking increases the risk of getting infected with the COVID-19 virus?
4. Do you think that smoking increases the risk of a severe course of the COVID-19 disease?
5. Do you think that if you succeed in quitting smoking now, you will maintain abstinence after the pandemic is over?
6. Do you think that if you fail to quit smoking now, you will try again after the pandemic is over?

In the period between 15 April 2020 and 31 May 2021, there were 5075 calls to the National Quitline. Answers to specific questionnaire regarding the COVID-19 pandemic were provided by 4072 callers (80%), comprising a group that then underwent thorough analysis. A total of 1520 callers (37%) declared that the COVID-19 pandemic impacted their decision of quitting smoking, prompting additional questions to be asked of them. Some missing values in answers to particular questions occurred, but without any substantial impact on the overall analysis (missing values for particular questions are included in the tables). We focused on attitudes and approaches to smoking cessation and the COVID-19 pandemic, especially its impact on smokers’ decision to quit, stratified by smoking status (heavy smoker/not heavy smokers) and respondent’s gender. Due to high differences between genders in smoking prevalence [12] as well as in lung cancer mortality in Poland [13], we decided to present our data separately for men and women. This approach may contribute to better understanding the differences between these two groups and what seems to be crucial for development of more effective gender-tailored health policy actions in the future.

Heavy smoking status was defined as smoking more than 20 cigarettes/day and smoking the first cigarette within the first 30 min after awakening. Not heavy smokers were defined as smoking less than 20 cigarettes/day or smoking the first cigarette after more than 30 min after awakening.

Calculations were performed using Microsoft Excel software version 16.16.27 (201012). *p*-values for the difference between proportions (between particular groups) were calculated with a Z-test. Significance level was set at α = 0.05.

### Limitations of the Study

The obtained sample is not representative of the whole country’s population. Moreover, interviews were conducted during different periods of severity of the pandemic, which was not taken into consideration in this paper. Strength of the impact of this factor has been changing over time and could affect smokers differently.

## 3. Results

### 3.1. Characteristics of the Respondents

A great majority of callers in this study were young men, with the highest proportion being 28% (746) at the age of 15–19 years old and 20–29 years old—24% (652). Among women, a near-equal percentage of callers were distributed across four consecutive age groups: 15–19 years—17% (230), 20–29 years—19% (258), 30–39 years—17% (236), and 40–49 years—18% (243). The highest number of callers was observed among men inhabiting towns (<100 thousand)—31% (491)—and smaller cities (100–500 thousand)—30% (477). In women, the largest groups were observed among those inhabiting big cities (>500 thousand)—31% (208)—and towns (<100 thousand)—30% (204) (Table 1).

Most of the callers were active smokers. In the sample, 91% (2468) of men and 83% (1121) of women were current smokers; 96% (2143) of men wanted to quit smoking and the same declaration was made by 97% (1058) of female callers. Among both men and women, the highest proportion of the callers were smokers who had been exposed to tobacco smoke for between 1 to 10 years—56% (1228) for men and 45% (473) in women. Most of the group smoked between 10 and 20 cigarettes a day among men—48% (1243)—and less than 10 cigarettes a day in the women’s group—51% (628). A majority of the analyzed group started smoking between the ages of 15 and 19—70% of men (1539) and 58% (602) of women. A small majority of the callers needed to smoke shortly (5–30 min) after awakening—55% (1172) of men and 52% (537) of women. On the other hand, a much smaller proportion of men—26% (555)—and women—14% (148)—needed to wake at night to smoke a cigarette (Table 1). Regarding the status of heavy and not heavy smokers (described in Section 2), we identified 19% (397) of heavy smokers and 81% (1743) of not heavy smokers among men and 9% (93) of heavy smokers and 91% (932) of not heavy smokers among women.

**Figure 1:**
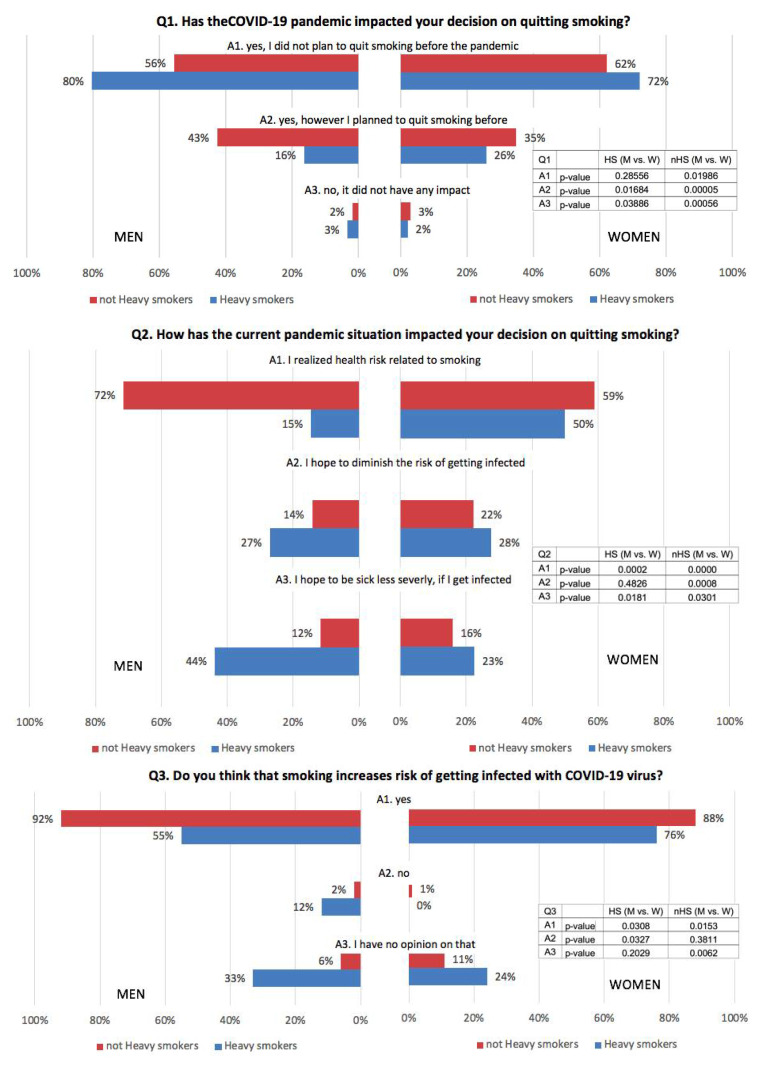

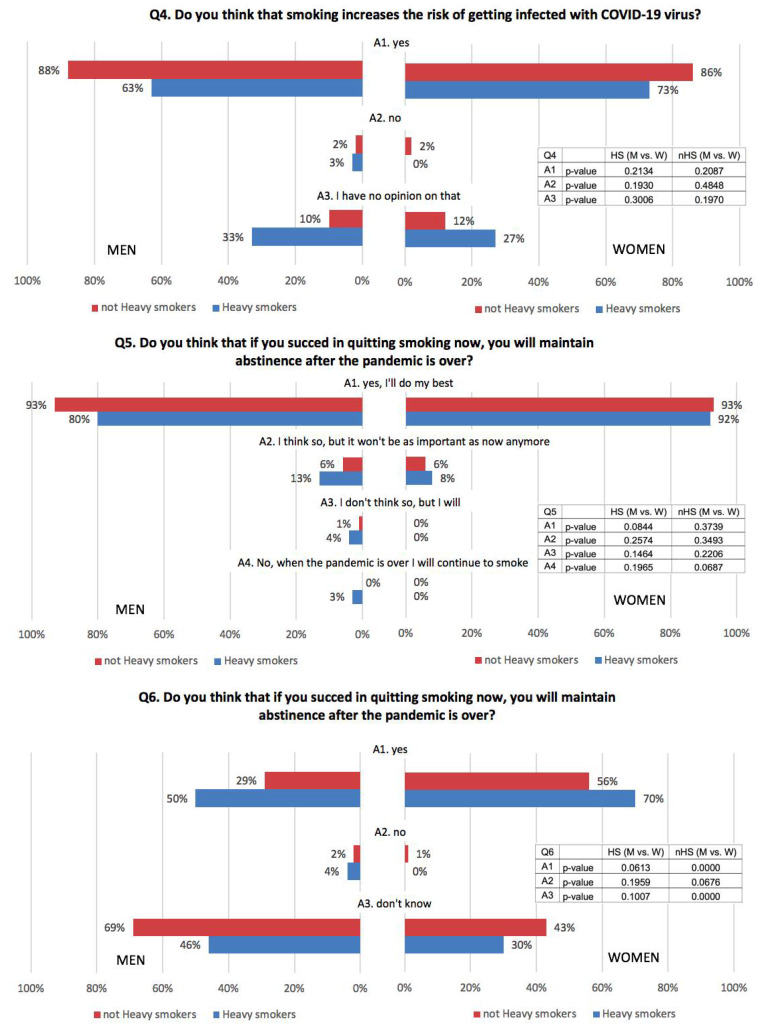
Impact of COVID-19 pandemic on decision of quitting smoking among heavy smokers and not heavy smokers.

Section 3.2 indicate the impact of COVID-19 pandemic on decision of quitting smoking—men (Figure 1, Table A1 in Appendix A), Section 3.3 indicate the impact of COVID-19 pandemic on decision of quitting smoking—women (Figure 1, Table A2 in Appendix B).

### 3.2. Impact of COVID-19 Pandemic on Decision of Quitting Smoking—Men

Regardless of smoking status, the vast majority of callers in the group of men (heavy/not heavy smokers) claimed that pandemic did not have any impact on their quitting decisions—80% (319) of heavy smokers and 56% (969) of not heavy smokers. In this case, smoking status significantly (*p* < 0.00) impacted this answer. Much fewer men—3% (13) of heavy smokers and 2% (31) of not heavy smokers—said that before the COVID-19 pandemic they did not consider quitting. In this particular subgroup, smoking status had no influence on the decision (*p* = 0.06); however, the small size of the subgroups in this case should be noted. As many as 72% (423) of not heavy smokers decided to after acknowledging the many tobacco-associated health risks. In contrast, only 15% (7) of heavy smokers considered this argument when making the decision on quitting. There was a significant difference between groups of heavy and not heavy smokers taking into account perception of higher health risk among smokers (*p* < 0.00). Our results also showed that for men who are heavy smokers, the most important factor for quitting was less severe course of the COVID-19 disease in case of infection—44% (21).

For heavy smokers, reduction of the infection risk was also a very important argument (the second-most frequently given answer)—27% (13). In the group of not heavy smokers, these percentages were at the level of 12% (69) and 14% (84), respectively. In both mentioned answers, there was a significant statistical difference between the groups of heavy and not heavy smokers (*p* < 0.00 and *p* = 0.02). In this context, the answers of heavy smokers regarding the perception of cigarette smoking as a factor increasing the risk of COVID-19 infection look very interesting, while as much as 33% (20) of heavy smokers claimed that they do not have an opinion on that. In the sample, 12% (9) reported that smoking does not increase the risk of infection with COVID-19. Finally, slightly over half of them—55% (40)—agreed that smoking increases the discussed risk. In comparison, only 6% (49) of the not heavy smokers claimed that they do not have an opinion on that, and just 2% (9) said that there is no increased risk. A high percentage of not heavy smokers—92% (693)—agreed that smoking increases the risk of getting infected with COVID-19 virus. Every answer in this question showed statistically significant differences between heavy smokers and not heavy smokers (*p* < 0.00). Looking at the next question, concerning the connection between smoking and COVID-19 severity, a similar trend in percentage proportions between heavy smokers and not heavy smokers is visible. Again, as much as 33% (49) of heavy smokers claimed that they do not have an opinion on that; 3% (2) stated that there is no such connection; and 63% (38) opined that smoking increases the risk of severe course of the COVID-19 disease. In the group of not heavy smokers, these percentages were, respectively, at the level of 10% (49), 2% (9), and 88% (431). However, in the case of the second answer (no connection), there was no statistically significant difference between heavy and not heavy smokers (*p* = 0.44). Considering abstinence after the pandemic, 80% (56) of heavy smokers and 93% (701) of not heavy smokers declared that they are going to maintain their decision anyway. Only 3% (2) of heavy smokers claimed that they are going to smoke again when the pandemic is over. None of the heavy smoker respondents provided such an answer (*p* < 0.00). In case of failure in quitting, heavy smokers declared more often to take future attempts to quit after the pandemic in comparison with not heavy smokers—50% (28) vs. 29% (142) (*p* < 0.00).

### 3.3. Impact of COVID-19 Pandemic on Decision of Quitting Smoking—Women

Regardless of the smoking status (heavy/not heavy smoker) among women, the highest proportion of the respondents claimed that the pandemic did not have any impact on their decision on quitting—72% (579) among heavy smokers and 62% (579) among not heavy smokers. Only 2% (2) of heavy smokers and 3% (28) of not heavy smokers said that COVID-19 was an important factor for quitting, and that they did not consider this before the pandemic. On the other hand, a much higher proportion of respondents agreed that while COVID-19 influenced their decision, they were already thinking about this before the outbreak of the pandemic—26% (24) of heavy smokers and 35% (325) of not heavy smokers. A lack of statistical significance (*p* > 0.05) indicated that the provided answers were not dependent on smoking status. Considering the influence of the current pandemic situation on quitting, the most important factor for both heavy and not heavy smokers was realizing the health risks related to smoking. The data show that 50% (20) of heavy smokers and 59% (219) of not heavy smokers claimed this as an important factor. Subsequently, the next most frequently given answer concerned the risk of infection—28% (82) of heavy smokers and 22% (82) of not heavy smokers wanted to diminish the risk of getting infected. Moreover, 23% (9) of heavy smokers and 16% (59) of not heavy smokers claimed that their decision on quitting was connected with hopes of a less severe course of the COVID-19 disease in case of infection. As much as 76% (19) of heavy smokers and 88% (298) of not heavy smokers agreed that smoking increases the risk of getting infected with the COVID-19 virus. Respectively, 73% (16) and 86% (229) admitted that they believe smoking increases the risk of severe course of the COVID-19 disease. None of the heavy smokers claimed that there is no connection between smoking and higher risk of infection and/or severe course of the disease. These percentages among not heavy smokers were, respectively, at the level of 1% (5) in case of infection risk and 2% (5) in case of the risk of severe course of the disease. In both questions, a surprisingly high proportion of women said that they do not have any opinion on that—24% (6) and 27% (6) of heavy smokers (smoking and higher risk of infection and severe course of the disease) as well as 11% (37) and 12% (32) of not heavy smokers. As much as 92% (23) of heavy smokers and 93% (316) of not heavy smokers declared that they want to continue abstinence when the pandemic is over. Respectively, 8% (2) and 6% (22) of women made the same declaration; however, it would not be as important for them as during the pandemic. Moreover, 70% (14) of heavy smokers and 56% (148) of not heavy smokers answered that in case of failure in quitting, they will try to quit again when the pandemic is over. On the other hand, as much as 30% (6) of heavy smokers and 43% (113) of not heavy smokers were not sure about that. In the analyzed group of women in almost all questions, the differences between heavy and not heavy smokers were not statistically significant at the level of α = 0.05. 

## 4. Discussion

According to 2020 Eurostat data, 32% of men and 20% of women in Poland smoked cigarettes (15 years old and older who reported that they currently smoke) [12]. Our study similarly had a higher proportion of the group who were men—about 2/3 of callers. These results are supported by other previous studies showing higher prevalence of heavy smokers among men in comparison to women (in our study 19% vs. 9%) [14,15,16].

Nicotine dependence is an officially classified disease (ICD-10: F17.200). The complex nature of the addiction, as well as the strong addictive character of nicotine, may lead to a distorted perception of the health risk connected with smoking. In our study, we investigated that, in most cases, the pandemic by itself did not have any impact on the decision on quitting (Figure 1). In contrast, however, we did find that health risks related to the pandemic and smoking had a substantial meaning for respondents in making such decision. Our study showed that a great majority of respondents, both men and women, agreed that smoking increases the risk of getting infected with the COVID-19 virus (Figure 1). Similar results were obtained in a study by Rigotti et al. In this research conducted among 694 current and daily smokers who were also COVID-19 patients, 68% of them believed that smoking is connected with higher risk of infection [17].

Moreover, results from a large-scale cross-sectional study on COVID-19 pandemic impacts on tobacco addiction conducted by Yang and Ma led to similar conclusions. The authors highlighted that health risks related to COVID-19 were a primary factor that contributes to quitting [18].

The results from the cross-sectional study by Al-Tammemi et al. (2424 participants) are also in line with our findings. Almost 72% of respondents claimed that smoking may lead to a severe course of the COVID-19 disease. Moreover, most of the participants presented the attitudes characterized by a negative approach to smoking during the pandemic [19].

Obtained results suggest that health risks related to the pandemic influenced the quitting decisions of the respondents (e.g., reduction of the risk of severe course of the disease in case of infection). Similar results were presented in the study by Nyman et al. [20], conducted among 1223 participants. The authors stressed that perceptions of the COVID-19 disease are among many other considerations made when deciding to attempt to quit smoking.

The results of the current study may also suggest that smokers are very receptive to information and arguments on health risk related to COVID-19 and smoking. A great majority of respondents—regardless of smoking status—combined their decision on quitting with a particular health argument (possible shortages in cigarette supplies and/or their accessibility were very rare answers). Similarly to this result, a study conducted by Pettigrew et al. in three countries (Australia, New Zealand, and the United Kingdom) among 1509 smokers showed that participants were very open and receptive to messages on COVID-19 and smoking risk prepared by the authors of the study [21].

In our study, we also investigated that after the pandemic, a vast majority of heavy smokers and not heavy smokers want to maintain abstinence. Moreover, in case of failure, they want to make another attempt to quit. These results suggest once again that the health threat related to the pandemic has a far-reaching influence on making quitting decisions. This phenomenon was also researched by Kayhan Tetik et al. [22]. The authors investigated smoking cessation success rates before and during the COVID-19 pandemic among 357 individuals. They found that smoking cessation in the investigated group before the pandemic was at the level of 23.7% and during that period was equal to 31.1%.

Obtained results showed that there were no statistically significant differences between compared fractions (heavy/not heavy smoker status) among women and in contrast to this, such differences were investigated in the great majority of cases among men. Possible explanation of this phenomenon comes from the characteristics of the heavy smokers. Since men usually present higher tobacco dependence, different features of heavy and not heavy smokers in this particular group may be more visible. Similar conclusions were confirmed in different studies, indicating significant differences by gender among heavy smokers [23]. Moreover, some of the studies confirmedthat smoking identity becomes more radicalized with the increasing number of smoked cigarettes, which may result in substantial differences between heavy and not heavy smokers within a group [24].

On the other hand, contrary to our findings, only one study conducted by Hwang et al. has shown that higher health risk connected with COVID-19 and smoking was not an important factor for smokers in their decision to quit [25]. However, this study was performed on a much smaller sample (36 participants), with the use of different methodology (focus groups), and also included participants using e-cigarettes and heated tobacco products.

## 5. Conclusions

For many years, a wide range of studies has suggested that health education campaigns appealing to fear are inappropriate and, ultimately, ineffective [26,27,28]. Our study results indicate that there is a need to reconsider this approach to some extent, particularly in the case of smokers. We investigated that the most important and convincing factors for smokers in the context of quitting smoking—regardless of their smoking status—were those related to fear for their own health and life. One of the recommendations could be to supplement warnings on tobacco products with information about health risks related to smoking and COVID-19.

Our results indicate that smokers are very receptive to communication concerning COVID-19 and smoking risk. This phenomenon can possibly be connected to the immediate potential health consequences of smoking and COVID-19 virus infection. Mentioned results may suggest that putting emphasis on arguments combined with short-term health consequences of smoking may result in better outcomes in smoking cessation. There is a need for further and constant education on tobacco health harm. Our results showed that an unusual and mass communication on health consequences may result in high effectiveness in smoking cessation. Additionally, such results might indicate that information and education on tobacco health harm should perhaps be limited to discuss one harm at a time. Such segmented communication will perhaps be more effective than information on several health consequences at once. 

Furthermore, it seems that smokers, when ill, are more willing to make a quitting attempt when they know it could improve their overall life chances. This is applicable also to chronic diseases such as cardiovascular diseases and cancer, where quitting smoking improves treatment outcomes. This message should be communicated in educational campaigns as it can promote smoking cessation among those who do not believe that smoking causes diseases and/or who already suffer from chronic conditions.

## Figures and Tables

**Table 1:** Characteristics of Polish National Quitline callers between 15 April 2020 and 31 May 2021.

Total Number of Records Analyzed: 4072	Men: 2716		Women: 1356	
	N	%	N	%
Age	2700		1349	
0–15	37	1%	14	1%
15–19	746	28%	230	17%
20–29	652	24%	258	19%
30–39	436	16%	236	17%
40–49	373	14%	243	18%
50–59	226	8%	141	10%
60+	230	9%	227	17%
Smoking status	2707		1354	
current smokers	2468	91%	1121	83%
in process of quitting smoking (abstinence < 3 mo)	202	7%	168	12%
ex-smokers	34	1%	59	4%
never smokers	3	0%	6	0%
Wants to quit smoking	2240		1086	
yes	2143	96%	1058	97%
no	82	4%	23	2%
hard to say	15	1%	5	0%
No. of cigarettes smoked a day	2575		1229	
less than 10	871	34%	628	51%
10–20	1243	48%	484	39%
21–40	354	14%	98	8%
more than 40	107	4%	19	2%
Length of exposure	2206		1051	
less than 1 year	67	3%	27	3%
1–10 years	1228	56%	473	45%
11–20 years	404	18%	233	22%
21–30 years	209	9%	95	9%
more than 30 years	298	14%	223	21%
Age of starting smoking	2185		1042	
less than 15 years	296	14%	93	9%
15–19 years	1539	70%	602	58%
20–29 years	316	14%	313	30%
30 years and later	34	2%	34	3%
Time from awakening to smoking first cigarette	2142		1025	
less than 5 min	469	22%	116	11%
5–30 min	1172	55%	537	52%
31–60 min	373	17%	277	27%
more than 60 min	128	6%	95	9%
Awakening at night to smoke	2132		1025	
yes	555	26%	148	14%
no	1577	74%	877	86%
Place of inhabitance	1579		678	
city > 500 thousand	401	25%	208	31%
city 100–500 thousand	477	30%	176	26%
town < 100 thousand	491	31%	204	30%
village	210	13%	90	13%

## Data Availability

Not applicable.

## Appendix A

**Table A1:** Smoking cessation attitudes presented among men by smoking status—absolute numbers, percentages, and statistical significance.

Total Number of Records Analyzed: 2140						
	Heavy Smokers		Not Heavy Smokers		*p*-Value	
	N	%	N	%		
	397	19%	1743	81%		
Has the COVID-19 pandemic impacted your decision on quitting smoking?						
N	397		1743			
yes, I did not plan to quit smoking before the pandemic	13	3%	31	2%	*p* =	0.06
yes, however I planned to quit smoking before	65	16%	743	43%	*p* <	0.00
no, it did not have any impact	319	80%	969	56%	*p* <	0.00
How has the current pandemic situation impacted your decision on quitting smoking?						
N	no of res. 48		no of res. 591			
I realized the health risk related to smoking	7	15%	423	72%	*p* <	0.00
I do not want to risk getting infected while going out to buy cigarettes	1	2%	4	1%	*p* =	0.29
I am afraid that the access to cigarettes will be limited	3	6%	4	1%	*p* <	0.00
I hope to diminish the risk of getting infected	13	27%	84	14%	*p* =	0.02
I hope to be less severely sick, if I get infected	21	44%	69	12%	*p* <	0.00
other	3	6%	7	1%	*p* =	0.01
Do you think that smoking increases the risk of getting infected with the COVID-19 virus?						
N	73		755			
yes	40	55%	693	92%	*p* <	0.00
no	9	12%	13	2%	*p* <	0.00
I have no opinion on that	24	33%	49	6%	*p* <	0.00
Do you think that smoking increases the risk of a severe course of the COVID-19 disease?						
N	60		489			
yes	38	63%	431	88%	*p* <	0.00
no	2	3%	9	2%	*p* =	0.44
I have no opinion on that	20	33%	49	10%	*p* <	0.00
Do you think that if you succeed in quitting smoking now, you will maintain abstinence after the pandemic is over?						
N	70		750			
yes, I will do my best	56	80%	701	93%	*p* <	0.00
I think so, but it will not be as important as now anymore	9	13%	44	6%	*p* =	0.02
I do not think so, but I will try	3	4%	5	1%	*p* <	0.00
no, when the pandemic is over I will continue to smoke	2	3%	0	0%	*p* <	0.00
Do you think that if you fail to quit smoking now, you will try again after the pandemic is over?						
N	56		489			
yes	28	50%	142	29%	*p* <	0.00
no	2	4%	11	2%	*p* =	0.54
do not know	26	46%	336	69%	*p* <	0.00

## Appendix B

**Table A2:** Smoking cessation attitudes presented among women by smoking status—absolute numbers, percentages, and statistical significance.

Total Number of Records Analyzed: 1025						
	Heavy Smokers		Not Heavy Smokers		*p*-Value	
	N	%	N	%		
	93	4%	932	44%		
Has the COVID-19 pandemic impacted your decision on quitting smoking?						
N	93		932			
yes, I did not plan to quit smoking before the pandemic	2	2%	28	3%	*p* =	0.64
yes, however I planned to quit smoking before	24	26%	325	35%	*p* =	0.08
no, it did not have any impact	67	72%	579	62%	*p* =	0.06
How has the current pandemic situation impacted your decision on quitting smoking?						
N	no of res. 40		no of res. 371			
I realized health risk related to smoking	20	50%	219	59%	*p* =	0.06
I do not want to risk getting infected while going out to buy cigarettes	0	0%	4	1%	*p* =	0.60
I am afraid that the access to cigarettes will be limited	0	0%	4	1%	*p* =	0.60
I hope to diminish the risk of getting infected	11	28%	82	22%	*p* =	0.50
I hope to be less severely sick, if I get infected	9	23%	59	16%	*p* =	0.36
other	0	0%	3	1%	*p* =	0.65
Do you think that smoking increases the risk of getting infected with the COVID-19 virus?						
N	25		340			
yes	19	76%	298	88%	*p* =	0.10
no	0	0%	5	1%	*p* =	0.54
I have no opinion on that	6	24%	37	11%	*p* =	0.05
Do you think that smoking increases the risk of a severe course of the COVID-19 disease?						
N	22		266			
yes	16	73%	229	86%	*p* =	0.09
no	0	0%	5	2%	*p* =	0.52
I have no opinion on that	6	27%	32	12%	*p* =	0.04
Do you think that if you succeed in quitting smoking now, you will maintain abstinence after the pandemic is over?						
N	25		340			
yes, I will do my best	23	92%	316	93%	*p* =	0.86
I think so, but it will not be as important as now anymore	2	8%	22	6%	*p* =	0.77
I do not think so, but I will try	0	0%	1	0%	*p* =	0.79
no, when the pandemic is over I will continue to smoke	0	0%	1	0%	*p* =	0.79
Do you think that if you fail to quit smoking now, you will try again after the pandemic is over?						
N	20		263			
yes	14	70%	148	56%	*p* =	0.23
no	0	0%	2	1%	*p* =	0.70
do not know	6	30%	113	43%	*p* =	0.26
